# Timing of norepinephrine initiation in patients with septic shock: a systematic review and meta-analysis

**DOI:** 10.1186/s13054-020-03204-x

**Published:** 2020-08-06

**Authors:** Yuting Li, Hongxiang Li, Dong Zhang

**Affiliations:** Department of Intensive Care Unit, The First Hospital of Jilin University, Changchun, 130021 Jilin China

**Keywords:** Timing, Norepinephrine initiation, Septic shock, Systematic review, Meta-analysis

## Abstract

**Background:**

The effect of the timing of norepinephrine initiation on clinical outcomes in patients with septic shock is uncertain. A systematic review and meta-analysis was performed to evaluate the impact of early and late start of norepinephrine support on clinical outcomes in patients with septic shock.

**Methods:**

We searched the PubMed, Cochrane, and Embase databases for randomized controlled trials (RCTs) and cohort studies from inception to the 1st of March 2020. We included studies involving adult patients (> 18 years) with septic shock. All authors reported our primary outcome of short-term mortality and clearly comparing early versus late norepinephrine initiation with clinically relevant secondary outcomes (ICU length of stay, time to achieved target mean arterial pressure (≥ 65 mmHg), and volume of intravenous fluids within 6 h). Results were expressed as odds ratio (OR) and mean difference (MD) with accompanying 95% confidence interval (CI).

**Results:**

Five studies including 929 patients were included. The primary outcome of this meta-analysis showed that the short-term mortality of the early group was lower than that of the late group (odds ratio [OR] = 0.45; 95% CI, 0.34 to 0.61; *P* < 0.00001; *χ*^2^ = 3.74; *I*^2^ = 0%). Secondary outcomes demonstrated that the time to achieved target MAP of the early group was shorter than that of the late group (mean difference = − 1.39; 95% CI, − 1.81 to − 0.96; *P* < 0.00001; *χ*^2^ = 1.03; *I*^2^ = 0%). The volume of intravenous fluids within 6 h of the early group was less than that of the late group (mean difference = − 0.50; 95% CI, − 0.68 to − 0.32; *P* < 0.00001; *χ*^2^ = 33.76; *I*^2^ = 94%). There was no statistically significant difference in the ICU length of stay between the two groups (mean difference = − 0.11; 95% CI, − 1.27 to 1.05; *P* = 0.86; *χ*^2^ = 0.85; *I*^2^ = 0%).

**Conclusions:**

Early initiation of norepinephrine in patients with septic shock was associated with decreased short-term mortality, shorter time to achieved target MAP, and less volume of intravenous fluids within 6 h. There was no significant difference in ICU length of stay between early and late groups. Further large-scale RCTs are still required to confirm these results.

## Key messages

- Early initiation of norepinephrine in patients with septic shock was associated with decreased short-term mortality, shorter time to achieved target MAP, and less volume of intravenous fluids within 6 h.
- There was no significant difference in ICU length of stay between early and late groups.
- More prompt and aggressive norepinephrine administration should be considered as part of initial resuscitation therapy for septic shock.

## Background

Septic shock is one of the most challenging problems in critical care medicine. With an increasing annual incidence in the developed world, mortality remains between 25 and 50% of those afflicted [1–3]. Patients with septic shock can be identified with a clinical construct of sepsis with persisting hypotension requiring vasopressors to maintain mean arterial pressure (MAP) ≥ 65 mmHg and having a serum lactate level > 2 mmol/L (18 mg/dL) despite adequate volume resuscitation [4]. The 2018 Surviving Sepsis Campaign (SSC) Bundle recommends administering broad-spectrum antibiotics, rapidly administering 30 ml/kg crystalloid for hypotension or lactate ≥ 4 mmol/L and applying vasopressors if the patient is hypotensive during or after fluid resuscitation to maintain MAP ≥ 65 mmHg within the first hour [5].

The pathophysiology of septic shock is complex and involves vasodilatation, relative and absolute hypovolemia, myocardial dysfunction, increased metabolic rate, and altered regional and microvascular blood flow [6–9]. Besides relative and absolute hypovolemia, decreased vascular tone is one of the major characteristics of septic shock causing hypotension [10]. Norepinephrine is both an alpha1- and beta1-agonist and is therefore able to increase vascular tone and contractility [11]. Recent guidelines recommend norepinephrine as the first-line vasopressor in septic shock [12].

So far, most studies have focused on the rational use of different types of vasopressors [13–15]. However, it is the timing of vasopressor therapy, rather than the specific agent, that appears to be crucial [16]. Studies comparing different agents have not clarified the optimal agents, and none have addressed the optimal timing [15, 17]. The present data show that the time from the onset of septic shock to initial norepinephrine administration is an important determinant of survival, but a recommendation on the timing to start norepinephrine support was not clearly stated [18].

Since the optimal timing of the initiation of norepinephrine remains unknown and whether the benefits or harm of norepinephrine introduction even preceding fluid resuscitation has not been still answered, we conducted a meta-analysis which extracted results from published randomized controlled trials (RCTs) and cohort studies to evaluate the impact of early and late start of norepinephrine support on clinical outcomes in patients with septic shock.

## Methods

This systematic review and meta-analysis is reported according to the Preferred Reporting Items for Systematic Reviews and Meta-Analyses (PRISMA) guidelines [19]. Ethical approval was not necessary for this study because it was a review of the published literature.

### Search strategy

**Fig. 1:**
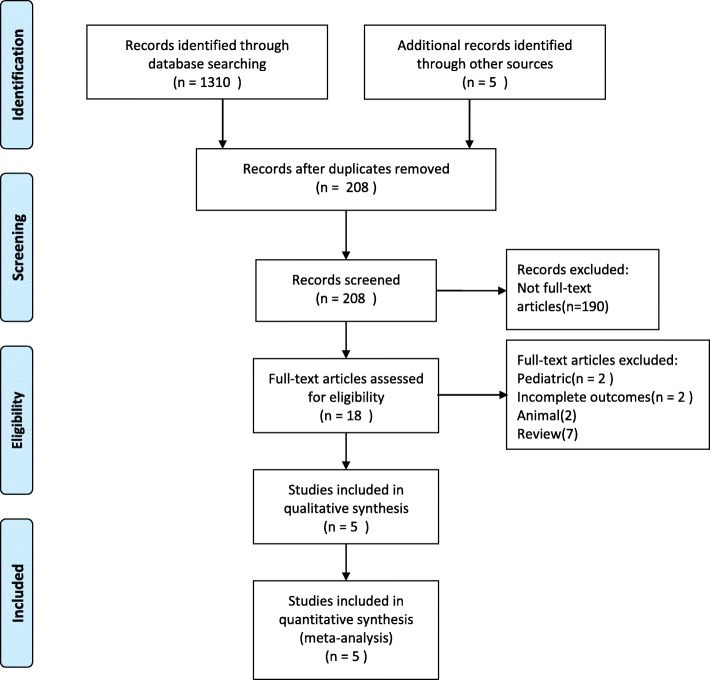
Flow chart of literature selection

We searched the PubMed, Cochrane, and Embase databases for studies from inception to the 1st of March 2020 using the following search terms: timing, time, early, earlier, delay, late, initiation, start, norepinephrine, vasopressor, and septic shock. The search was slightly adjusted according to the requirements of the different databases. The authors’ personal files and reference lists of relevant review articles were also reviewed. The flow chart of the search strategies is summarized in Fig. 1.

### Types of outcome measures

The primary outcome was short-term mortality; short-term mortality included hospital mortality, 28-day mortality, and 30-day mortality. Secondary outcomes were intensive care unit (ICU) length of stay, time to achieved target MAP (≥ 65 mmHg), and volume of intravenous fluids within 6 h. Weighted means were calculated based on the number of patients in each study.

### Study selection

The inclusion criteria were as follows: (1) RCTs as well as prospective and retrospective cohort studies; (2) adult patients (> 18 years) with septic shock, septic shock was classified according to the current Third International Consensus Definitions for Sepsis and Septic Shock (Sepsis 3.0), which considers the presence of suspected infection accompanying organ dysfunction, the use of vasopressors, MAP < 65 mmHg, and lactate levels > 2 mmol/L [4]; (3) all authors reported our primary outcome of short-term mortality; (4) and clearly comparing early versus late norepinephrine initiation with clinically relevant secondary outcomes. We excluded studies [20, 21] without clear comparisons of the outcomes. In addition, we excluded review articles and studies about pediatric or animal.

### Quality assessment

Two reviewers (YL and HL) independently performed quality assessment. The quality of studies was assessed using the Cochrane Collaboration’s tool for RCTs [22], and the Newcastle-Ottawa Scale (NOS) was used for cohort studies [23]. The specific elements to minimize bias of RCTs were (1) randomization sequence (selection bias), (2) allocation concealment (selection bias), (3) blinding of study personnel and participants (performance bias), (4) blinding of outcome assessors (performance bias), (5) complete reporting of data without arbitrarily excluded patients and with low to minimal loss to follow-up (attrition bias), (6) selective reporting bias, and (7) other sources of bias. Satisfactory performance, unclear performance, and unsatisfactory performance of each domain from the tool are denoted by green, yellow, and red colors respectively. The risk of bias summary for included RCTs is presented in Supplement 1; the risk of bias graph for included RCTs is presented in Supplement 2.

NOS allocates a maximum of 9 points according to the quality of the selection, comparability, and outcomes of the cohort study populations. Study quality was defined as poor (0–3), fair (4–6), or good (7–9). The quality of the included cohort studies is presented in Table 1.

**Table 1:** Quality of the included cohort studies (The Newcastle-Ottawa Scale)

Study	Selection				Comparability	Outcome			
	Representativeness of the exposed cohort	Selection of the non-exposed cohort	Ascertainment of exposure	Demonstration that outcome of interest was not present at the start of the study	Comparability of cohorts on the basis of the design or analysis	Assessment of outcome	Was follow-up long enough for outcomes to occur	Adequacy of follow-up of cohorts	Total score
**Bai et al. 2014** [24]	☆	☆	☆	☆	☆☆	☆	☆	☆	9
**Colon Hidalgo et al. 2020** [25]	☆	☆	☆	☆	☆☆	☆	☆	☆	9
**Ospina-Tascón et al. 2020** [26]	☆	☆	☆	☆	☆☆	☆	☆	☆	9

### Statistical analysis

Statistical analyses were performed using Review Manager version 5.3 (RevMan, The Cochrane Collaboration, Oxford, UK). Odds ratio (OR) with 95% confidence intervals (CI) was calculated for dichotomous variables. As to the continuous variables, mean difference (MD) and 95% CI were estimated as the effect result. A random-effects model was used to pool studies with significant heterogeneity, as determined by the chi-squared test (*P* < 0.10) and inconsistency index (*I*^2^ ≥ 50%) [27]. Some of the selected continuous variables were represented by the median (interquartile range). We calculated their mean and standard deviation according to the sample size with a calculator [28] and then performed a meta-analysis. A *P* value < 0.05 was set as the threshold of statistical significance. To reduce bias, we performed a subgroup analysis of RCTs and cohort studies.

## Result

### Study characteristics

The search strategy identified 1315 studies, and the data were from 2 RCTs and 3 cohort studies comprising 929 patients (Table 2) [24–26, 29, 30]. The characteristics of the included studies are shown in Table 2. A total of 5 eligible studies were published between 2014 and 2020. Among these studies, one study was conducted in China, one study was conducted in Thailand, one study was conducted in Egypt, one study was conducted in the USA, and one study was conducted in Colombia. All of these studies were single-center studies. The definitions of early and late norepinephrine group in studies included in the meta-analysis are outlined in Table 3.

**Table 2:** The basic characteristics of studies included in the meta-analysis

Author	Year	Country	Study period	Study design	No. of patients		
					Total	Early group	Late group
Bai et al. [24]	2014	China	Jan. 2011–Dec. 2012	Single center, retrospective cohort study	213	86	127
Permpikul et al. [29]	2019	Thailand	Oct. 2013–Mar. 2017	Single center, RCT	310	155	155
Elbouhy et al. [30]	2019	Egypt	Jan. 2017–Dec. 2018	Single center, RCT	101	57	44
Colon Hidalgo et al. [25]	2020	USA	Jan. 2017–Jul. 2017	Single center, retrospective cohort study	119	76	43
Ospina-Tascón et al. [26]	2020	Colombia	Jan. 2015–Feb. 2017	Single center, prospective cohort study	186	93	93

**Table 3:** Definitions of early and late norepinephrine group in studies included in the meta-analysis

Study	Early group	Late group
Bai et al. 2014 [24]	Time from the onset of septic shock to initial norepinephrine administration < 2 h	Time from the onset of septic shock to initial norepinephrine administration ≥ 2 h
Permpikul et al. 2019 [29]	Median time from emergency room arrival to norepinephrine administration was 93 min	Median time from emergency room arrival to norepinephrine administration was 192 min
Elbouhy et al. 2019 [30]	Patients received initial resuscitation as simultaneous administration of crystalloid fluids (the target was 30 mL/kg) together with norepinephrine infusion in a starting dose of 5 μg/min administered in an external jugular peripheral cannula	Patients’ resuscitation included crystalloid fluids (the target was 30 mL/kg) and immediate ICU transfer where the norepinephrine infusion was administered only to patients with mean arterial pressure < 65 mmHg after fluids resuscitation via a central venous catheter
Colon Hidalgo et al. 2020 [25]	The time when vasopressors were initiated ≤ 6 h	The time when vasopressors were initiated > 6 h
Ospina-Tascón et al. 2020 [26]	Vasopressor support initiated within the next hour or even before the first fluid load with resuscitative intention (FRLoad)	Patients in whom vasopressor support was started > 1 h after the FRLoad

### Primary outcome

**Fig. 2:**
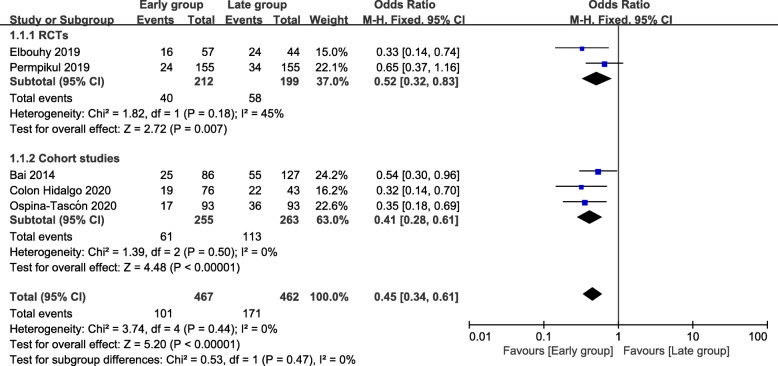
Forest plot for short-term mortality

**Fig. 3:**
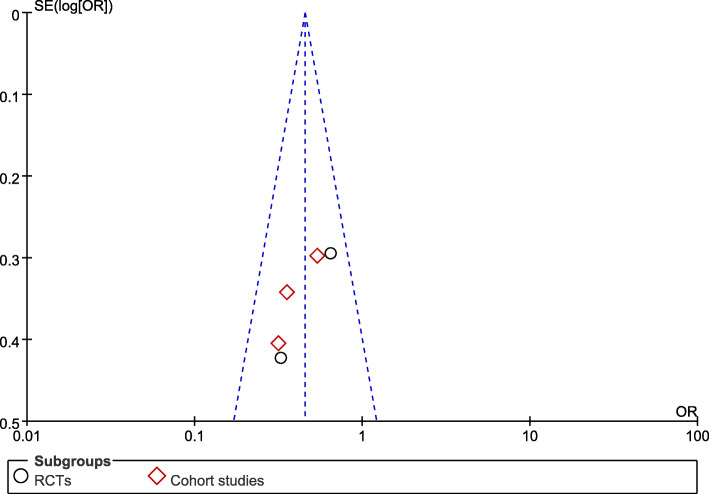
Funnel plot for short-term mortality

A total of five studies including 929 patients were included, and the short-term mortality was about 29.3% (101/467 in the early group and 171/462 in the late group). The short-term mortality of the early group was lower than that of the late group (odds ratio [OR] = 0.45; 95% CI, 0.34 to 0.61; *P* < 0.00001; *χ*^2^ = 3.74; *I*^2^ = 0%) (Fig. 2). A funnel plot was used to assess the publication bias (Fig. 3).

### Secondary outcomes

#### ICU length of stay

**Fig. 4:**
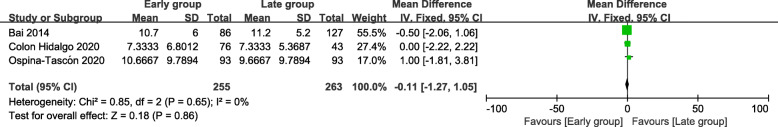
Forest plot for ICU length of stay

Three of included studies were analyzed to assess the ICU length of stay (day). There was no statistically significant difference in the ICU length of stay between the two groups (mean difference = − 0.11; 95% CI, − 1.27 to 1.05; *P* = 0.86; *χ*^2^ = 0.85; *I*^2^ = 0%) (Fig. 4).

#### Time to achieved target MAP

**Fig. 5:**
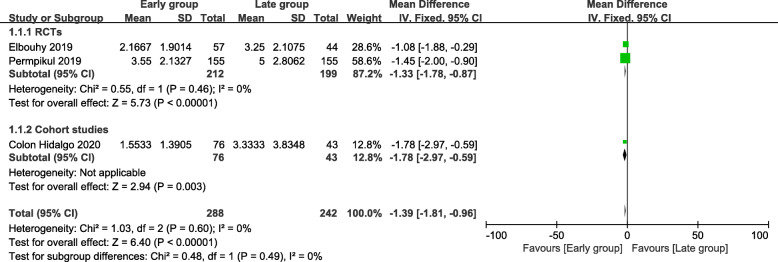
Forest plot for time to achieved target MAP

Three of the included studies were analyzed to assess the time to achieved target MAP (hour). The time to achieved target MAP of the early group was shorter than that of the late group (mean difference = − 1.39; 95% CI, − 1.81 to − 0.96; *P* < 0.00001; *χ*^2^ = 1.03; *I*^2^ = 0%) (Fig. 5).

#### Volume of intravenous fluids within 6 h

**Fig. 6:**
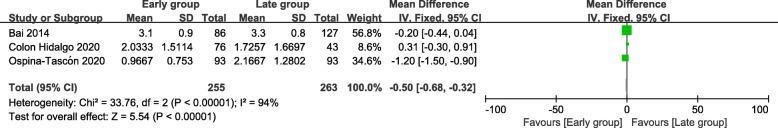
Forest plot for volume of intravenous fluids with 6 h

Three of included studies were analyzed to assess the volume of intravenous fluids within 6 h (L). The volume of intravenous fluids within 6 h of the early group was less than that of the late group (mean difference = − 0.50; 95% CI, − 0.68 to − 0.32; *P* < 0.00001; *χ*^2^ = 33.76; *I*^2^ = 94%) (Fig. 6).

## Discussion

This systematic review and meta-analysis of five studies including 929 patients compared early and late norepinephrine initiation in patients with septic shock. We found that the overall short-term mortality was about 29.3%, and the short-term mortality of the early group was lower than that of the late group. Secondary outcomes showed that the time to achieved target MAP of the early group was shorter than that of the late group. The survival benefit of early and effective resuscitation is confirmed in septic shock patients [31]. This meta-analysis demonstrated that the significant mortality benefit with early initiation of a norepinephrine is likely secondary to earlier achievement and maintenance of adequate perfusion pressures, preventing the onset and/or progression of organ dysfunction [32]. This is also in line with another study suggesting that shorter hypotension times are associated with better outcomes in septic shock [33]. The patients with septic shock receiving vasopressors early or late depend on the patient’s response to initial fluid resuscitation and the judgment of the physicians. If clinicians delay starting vasopressors because of a lack of critical care bed availability, then they probably should not delay. Managing a patient on a general ward, without vasopressors, hoping that in time blood pressure will improve and thus not require critical care, may lead to worse outcomes for patients [34]. Since the timing to start norepinephrine is crucial, one of the recommendations was to administer norepinephrine in the initial phase of septic shock even when hypovolemia is not completely corrected by fluid administration [35]. In addition, a low value of diastolic arterial pressure (e.g., < 40 mmHg) is strongly suggestive of a markedly depressed arterial tone and should prompt initiation of norepinephrine urgently [36].

Secondary outcomes demonstrated that the volume of intravenous fluids within 6 h of the early group was less than that of the late group. Fluid overload is a common complication during septic shock resuscitation. Two recent studies also showed that early use of norepinephrine is associated with less fluid administration and improved outcomes [37, 38]. Positive cumulative fluid balance is an independent factor of mortality in septic shock patients: the higher the positive fluid balance, the poorer the outcome [39–41]. A recent large cohort study of 23,513 patients with severe sepsis and septic shock showed that administration of more than 5 L of fluid during the first day is associated with a significantly increased risk of death [42]. The mechanisms by which excessive fluid administration may worsen outcome include peripheral tissue edema with risks of multiple organ dysfunction, pulmonary edema with risks of profound hypoxemia, degradation of endothelial glycocalyx [43] with risks of increased vascular permeability, marked increase in venous pressures with risks of decrease in organ perfusion pressure [44], and hemodilution [45]. Early use of norepinephrine decreases the use of fluid replacement, possibly by constricting the dilated vascular bed, and shortens resuscitation duration [46]. In addition, our meta-analysis showed that there was no statistically significant difference in the ICU length of stay between the two groups. Delay in vasopressor initiation was not predictive of ICU length [47].

Early norepinephrine use in septic shock can increase cardiac output through an increase in cardiac preload and/or contractility and improve microcirculation and tissue oxygenation [36]. However, the use of vasopressors is not without consequences. Risk of well-known adverse reactions, such as arrhythmias, may have been increased for patients with prolonged exposure to vasopressors, potentially adding to the increased mortality rate. Another main argument is that high doses of exogenous norepinephrine may have deleterious consequences such as myocardial cell injury, oxidative stress, and alteration of sepsis-associated immunomodulation [48]. Splanchnic hypoperfusion is also an important concern when norepinephrine is given early. No matter what, norepinephrine remains the primary vasopressor in septic shock, and the existing evidence suggests that it remains a safe and effective first-line medication for septic shock.

At present, there is no uniform definition of “early” or “late” norepinephrine initiation in patients with septic shock, and the five studies included in our meta-analysis have different definitions of the early and late groups. We listed a table (Table 3) to illustrate each author’s definition of the early and late groups. The recent Hour-1 Bundle supported by the SSC recommends starting vasopressors within the first hour of resuscitation if initial fluid loading does not restore minimum MAP [5]. Indeed, norepinephrine infusion can be safely started before ICU admission, even in intermediate care without intensivist supervision. Delays in initiation of vasopressor therapy following the first documentation of hypotension in septic shock are modestly associated with increased specific organ failure and mortality risk. We now need a large multicenter phase 3 RCT of early norepinephrine initiation powered for mortality and organ dysfunction. In a word, early may be better.

This meta-analysis and the five included studies have several characteristics. First, the effect of the timing of norepinephrine initiation on short-term mortality is different in the five included studies. Therefore, the meta-analysis of different studies with different conclusions adds to the significance of this study. Second, although the primary outcomes of the five studies are inconsistent, the secondary outcomes, including the time to achieved target MAP and ICU length of stay, are consistent. This will reinforce the secondary outcomes of this meta-analysis.

Our meta-analysis has several limitations. First, the number of included studies is small. Further randomized clinical studies should be conducted in order to confirm the results. Second, many of the secondary outcomes such as ICU length of stay, time to achieved target MAP, or volume of intravenous fluids within 6 h were not included in all of the studies examined in this meta-analysis. Third, organ dysfunction is also a very important clinical outcome. However, few included studies had shown this data. Fourth, although we had performed a subgroup analysis of RCTs and cohort studies, there was still substantial heterogeneity among the included studies. Very heterogeneous populations were included in both randomized and observational studies. In addition, inclusion/exclusion criteria and comorbidities were widely different among included studies which supposed a limitation to interpret results. Therefore, our findings should be interpreted with caution.

## Conclusion

Early initiation of norepinephrine in patients with septic shock was associated with decreased short-term mortality, shorter time to achieved target MAP, and less volume of intravenous fluids within 6 h. There was no significant difference in ICU length of stay between early and late groups. These results suggest that more prompt and aggressive norepinephrine administration should be considered as part of initial resuscitation therapy for septic shock. Further large-scale RCTs are still required to confirm these results.

## Supplementary information

**Additional file 1: Supplement 1.** Risk of bias summary: review authors’ judgements about each risk of bias item for each included RCTs.

**Additional file 2: Supplement 2.** Risk of bias graph: review authors’ judgements about each risk of bias item presented as percentages across all included RCTs.

## Data Availability

All data generated or analyzed during this study are included in this published article.

## Abbreviations

MAP: Mean arterial pressure
SSC: Surviving Sepsis Campaign
RCTs: Randomized controlled trials
ICU: Intensive care unit
PRISMA: Preferred Reporting Items for Systematic Reviews and Meta-Analyses
NOS: Newcastle-Ottawa Scale
OR: Odds ratio
MD: Mean difference
CI: Confidence interval
