# Supplementary material for: Timing of norepinephrine initiation in patients with septic shock: a systematic review and meta-analysis
Source: Crit Care. 2020 Aug 6;24:488. doi: 10.1186/s13054-020-03204-x (PMC7409707; doi:10.1186/s13054-020-03204-x)
Supplement: Supplementary file 1 — Additional file 1: Supplement 1. Risk of bias summary: review authors’ judgements about each risk of bias item for each included RCTs. [file 13054_2020_3204_MOESM1_ESM.pdf]

	Random sequence generation (selection bias)	Allocation concealment (selection bias)	Blinding of participants and personnel (performance bias)	Blinding of outcome assessment (detection bias)	Incomplete outcome data (attrition bias)	Selective reporting (reporting bias)	Other bias
Elbouhy 2019	+	+	+	+	+	+	?
Permpikul 2019	+	+	+	+	+	+	?
