# Supplementary figures and images for: Timing of norepinephrine initiation in patients with septic shock: a systematic review and meta-analysis
Source: Crit Care. 2020 Aug 6;24:488. doi: 10.1186/s13054-020-03204-x (PMC7409707; doi:10.1186/s13054-020-03204-x)

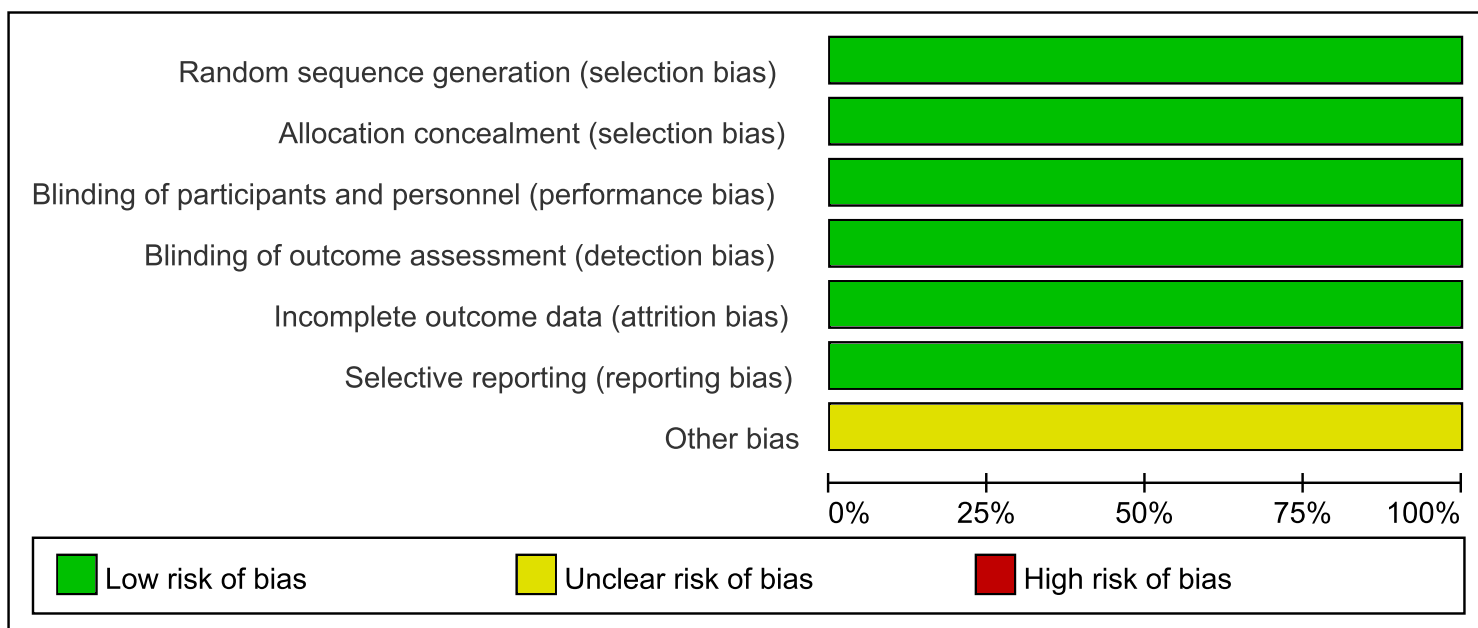

Supplement: Supplementary file 2 — Additional file 2: Supplement 2. Risk of bias graph: review authors’ judgements about each risk of bias item presented as percentages across all included RCTs. [file 13054_2020_3204_MOESM2_ESM.pdf]
